# Alternative Lengthening of Telomeres in Pediatric Cancer: Mechanisms to Therapies

**DOI:** 10.3389/fonc.2019.01518

**Published:** 2020-01-21

**Authors:** Thomas Kent, Deanne Gracias, Samuel Shepherd, David Clynes

**Affiliations:** ^1^MRC Molecular Haematology Unit, MRC Weatherall Institute of Molecular Medicine, John Radcliffe Hospital, University of Oxford, Oxford, United Kingdom; ^2^Department of Oncology, MRC Weatherall Institute of Molecular Medicine, John Radcliffe Hospital, University of Oxford, Oxford, United Kingdom

**Keywords:** alternative lengthening of telomeres, ATRX, break induced replication, Rad52, telomeres, R-loops, G-quadruplexes

## Abstract

Achieving replicative immortality is a crucial step in tumorigenesis and requires both bypassing cell cycle checkpoints and the extension of telomeres, sequences that protect the distal ends of chromosomes during replication. In the majority of cancers this is achieved through the enzyme telomerase, however a subset of cancers instead utilize a telomerase-independent mechanism of telomere elongation—the Alternative Lengthening of Telomeres (ALT) pathway. Recent work has aimed to decipher the exact mechanism that underlies this pathway. To this end, this pathway has now been shown to extend telomeres through exploitation of DNA repair machinery in a unique process that may present a number of druggable targets. The identification of such targets, and the subsequent development or repurposing of therapies to these targets may be crucial to improving the prognosis for many ALT-positive cancers, wherein mean survival is lower than non-ALT counterparts and the cancers themselves are particularly unresponsive to standard of care therapies. In this review we summarize the recent identification of many aspects of the ALT pathway, and the therapies that may be employed to exploit these new targets.

## Introduction

Achieving replicative immortality is a hallmark of cancer and is essential for cancer proliferation (1). Due to the end replication problem, wherein DNA polymerases fail to replicate the distal ends of chromosomes, chromosomal DNA is progressively shortened through each round of division, ultimately threatening genomic stability (2). Humans, like many other species, have evolved protective repetitive sequences called telomeres, nucleoprotein structures that act as a buffer to the end replication problem as well as a barrier to the recognition of DNA ends by DNA repair machinery (3). These sequences range from 3 to 12 kb in humans and consist of TTAGGG_n_ repeats interspersed with a group of telomere proteins called the Shelterin complex (4). The Shelterin complex, consisting of TRF1, TRF2, POT1, TIN1, TPP1 and RAP1, acts primarily to protect and aid in the structuring of telomeres (5). In this regard, the Shelterin complex works in concert to promote the formation of loop structures named t-loops from the overhanging single-stranded G-rich DNA (ssDNA) that, in turn, prevents the ssDNA ends from being recognized as a double strand break (DSB) (6).

The progressive shortening of telomeres, by ~200 bp per cell division, acts as a cellular clock that ensures turnover of cells that have gone through many rounds of division and potentially acquired a large number of mutations. At the end of this process, termed the Hayflick limit, telomeres reach critically short lengths and trigger cellular senescence (7).

Some cells, namely long-lived cells such as stem and early progenitor cells, require maintenance of these telomeric sequences to allow for long term survival. These cells use the telomere elongating enzyme telomerase to maintain telomere length. Telomerase itself is a combination of two principle components, the 1,132 amino acid telomerase reverse transcriptase (TERT) and an associated telomerase RNA molecule (TERC) (8, 9). Together, telomerase progressively adds telomeric sequence to the end of telomeres (7). The expression and activity of telomerase is tightly controlled, limiting its use in normal dividing cells (10).

Many cancers, 85–97%, utilize this natural telomere extension mechanism and use it to maintain their own telomeres, allowing them to evade telomere crisis (11). A minority of cancers, however, have established telomerase-independent mechanisms of telomere elongation. These cancers, collectively, are referred to as the Alternative Lengthening of Telomeres (ALT) cancers (12). ALT cancers present an exciting avenue of study due to their unique nature. In this review we will explore recent literature that is aimed at understanding the mechanism by which ALT arises, as well as comment on emerging or potential therapies targeted at ALT cancers.

## What is ALT?

Despite being present in a minority of cancers overall, the prevalence of ALT in cancers is not uniform, with cells of mesenchymal origin being more likely to rely on ALT for telomere elongation (13). Indeed, certain cancers, such as osteosarcomas and cancers of the central nervous system, have rates of ALT positivity approaching 90%, eluding to possible mechanistic reasons for ALT development (11). The most likely cause of this distribution is that, in contrast to cells of epithelial origin, cells of mesenchymal origin are more likely to have more stringently regulated telomerase expression, reducing the potential for telomerase-mediated telomere maintenance (13). ALT cancers can be particularly hard to treat effectively, in part due to their distribution, often ruling out early resection and their unique mechanism of maintenance leaving them insensitive to therapies that target telomerase.

In contrast to telomerase mediated telomere extension ALT is generally considered to be a form of aberrant telomeric recombination constituting conservative replication (14, 15). Along with a lack of reliance on telomerase, ALT is characterized by a number of markers (16). The first, the presence of extrachromosomal circular DNA is often regarded as the gold standard of ALT diagnosis. This extrachromosomal DNA is in fact partially double stranded telomeric DNA, that is either C-rich or G-rich and are termed C- and G-circles, respectively. In the diagnosis of ALT, rolling circle amplification of C-circles allows for rapid quantification of telomeric circles, which correlate well with ALT positivity (17). Two further characteristics include telomere sister chromatid exchanges (tSCEs) and heterogenous telomere lengths. ALT cancers also display increased replicative stress and telomeric DNA damage induced foci (TIFs), a potential driver of ALT generation (18, 19). Finally, ALT cells display characteristic telomere clustering and localization to promyelocytic leukemia (PML) bodies, forming structures named ALT-associated PML bodies (APBs) (20). Recent work has identified two possible new markers of ALT: mitotic DNA synthesis (MiDAS) and upregulation of the long non-coding Telomeric Repeat-containing RNA (TERRA) (21, 22).

## The Mutational Landscape of ALT

Somatic mutations in the α-thalassemia/mental retardation syndrome X-linked proteins (ATRX) and the death domain-associated protein (DAXX) chromatin remodeling complex are by far the most common mutations in ALT and are highly correlated with ALT development (23). Nevertheless, mutations in other proteins have been described in ALT that are believed to be involved in, or may lead to the development of the ALT phenotype. These include mutations in histone H3.3, SMARCAL1, and IDH1 (24–26). Additional correlated mutations may indeed exist, however due to the lack of routine testing for ALT in clinic, many ALT cancers are likely never fully characterized.

## The Role of ATRX in ALT Development

Recent work has improved our understanding of the underlying mechanism of ALT, however, despite this, it is still not clear as to the exact process by which ALT development occurs. Due to the near universal loss of the SWI/SNF protein ATRX in ALT cancer, and the ability of ATRX to suppress markers of ALT in a DAXX dependent manner, it would appear that loss of ATRX is a key factor in the development of ALT (27–29). Indeed, one recent study showed that, in certain cell lines, markers of ALT could be triggered upon loss of ATRX alone (28). This observation is in contrast to the majority of cases where ATRX loss alone is insufficient to trigger ALT and raises interesting questions as to the other underlying factors present in these cells. Thus, ATRX is not the silver bullet of ALT development one might expect. It is, therefore, important to consider additional causal factors in the development of ALT.

Although one of ATRX's primary roles is in the ATRX-DAXX-H3.3 histone deposition pathway, wherein ATRX promotes the deposition of histone variant H3.3 into telomeres, ATRX has also been found to be a potent regulator of histone variant macroH2A incorporation into chromatin (30). It has recently been shown that the interaction between ATRX and macroH2A1.2 additionally acts in a protective capacity to maintain fork stability during acute replication stress (31). In ALT cells lacking ATRX, despite telomeric macroH2A1.2 being generally enriched, macroH2A1.2 is transiently lost during replication stress, leading to increased fork collapse, a potential driver of the ALT pathway (32). In concert with this, loss of ATRX additionally permits the binding of the alternative macroH2A isoform, macroH2A1.1, to the poly(ADP-ribose) polymerase tankyrase 1 in a DNA damage response dependent manner, preventing its localization to telomeres and resolution of cohesion. The result of this persistent telomere cohesion is an increase in tSCEs and a simultaneous suppression of excessive non-sister telomeric recombination that is detrimental to ALT cell growth (33).

Many of the proteins mutated in ALT cancers are related, in part, to replication stress or DNA break repair deficiency. ATRX has previously been shown to be both involved in both of these processes, with loss of ATRX being associated with increases in replicative stress, fork stalling, fork restart, and fork protection (34–36). A recent analysis of proteins recruited to common fragile sites (CFS) identified ATRX as a pivotal player in CFS stability upon induction of replicative stress, with ATRX being recruited to a subset of CFS with DAXX in a FANCD2 dependent manner (37).

Additionally, evidence exists for a role of ATRX downstream of fork collapse in DNA double strand break repair. Work in human glioblastomas has indicated that ATRX loss leads to a reduction in non-homologous end joining (NHEJ), and renders cells lacking ATRX sensitive to a number of DSB inducing agents and ionizing radiation (IR) (38). In contrast, Juhász et al. demonstrated no reduction in NHEJ efficiency in response to the loss of ATRX, and instead implicated ATRX in long tract DNA repair by homologous recombination (39). Additionally, the study suggested that ATRX-null cells were sensitive to both methyl methanesulfonate (MMS) and mitomycin C (MMC), an alkylating agent and DNA crosslinking agent, respectively. Despite the contrasting messages of these studies, both present potential therapies that could target ALT cancers.

## ALT in Glioma

ALT cancers are particularly common in cancers of the central nervous system, with rates as high as 63% (11). Mutations in the isocitrate dehydrogenase enzyme (IDH1), specifically the R132H mutation, almost exclusively occur in gliomas and other cancers of the central nervous system. To date, no extensive work has been performed to quantify the prevalence of IDH1 mutations across the spectrum of ALT cancers, however, previous literature has shown that these mutations correlate strikingly with ALT status in certain tumor types (26). Work by Mukherjee et al. investigated these mutations in the context of human ALT gliomas and found that IDH1 R132H mutations led to a consistent downregulation of a number of proteins, two of which being the Shelterin component RAP1, and the DNA damage repair protein XRCC1. The authors show that in the absence of ATRX, loss of both RAP1 and XRCC1 leads to an increase in ALT markers (26). Downregulation of RAP1 has previously been shown to lead to an increase in telomere dysfunction by end uncapping, however a previous study showed that following TALEN knockout RAP1 loss did not in itself lead to an increase in telomere dysfunction or tSCEs (40, 41). This lends credibility to the hypothesis proposed by the authors, that RAP1 loss along with a cooperating ATRX mutation are required to trigger the phenotype. XRCC1 loss, on the other hand, leads to a deficiency in microhomology-mediated end joining (MMEJ), a critical pathway for efficient repair of homologous repeat sequences (26). Along with a role in MMEJ, XRCC1 has additional roles in both base excision repair (BER) and nucleotide excision repair (NER) (42, 43). In both cases, loss of XRCC1 would lead to persistent ssDNA breaks, which following replication could present a barrier to replication and eventual formation of a DSB.

## ALT Requires Telomeric Heterochromatin

In contrast to much of the genome, which is euchromatic, telomeres are naturally heterochromatic structures, a term used to describe condensed DNA that bares a variety of histone modifications. The predominant marker at heterochromatic regions is the histone H3 lysine 9 trimethylation (H3K9me3), which is in turn bound by a number of proteins including ATRX and heterochromatin protein 1 (HP1) (44–46). Outside of telomeric heterochromatin, at the pericentromeres, heterochromatin formation is promoted by two histone methyltransferases, the suppressor of variegation 3–9 homologs (SUV39H1 and SUV39H2), which are collectively known as SUV39H. Loss of Suv39h itself leads to formation of many of the markers of ALT, including APBs and tSCEs, and it was therefore assumed that SUV39H acted as the major propagator of telomeric heterochromatin, and that SUV39H protected telomeres from ALT (47, 48). In this regard, ALT telomeres display reduced condensation and decreased H3K9me3 marks, which lead to a reduction in chromatin compaction (49).

Recent literature, however, has challenged this idea, suggesting that, in fact, ALT telomeres are enriched for H3K9me3, and non-ALT telomeres are largely euchromatic (50). Additionally, work has shown that, contrary to long standing dogma, telomeric heterochromatin formation is instead mediated through the H3K9 methyltransferase activity of SET Domain Bifurcated 1 protein (SETDB1), and this heterochromatin formation drives the development of ALT. In this work the authors demonstrate that SETDB1 loss leads to a reduced recruitment of ALT-related factors to telomeres, and a reduction in ALT markers including C-circles and APBs, and that, therefore, heterochromatin formation at telomeres is required for ALT (51).

Together these data suggest a potentially pivotal role of chromatin landscape in the development and maintenance of ALT, the specifics of which may guide a number of cellular processes. Additionally, the observation of ALT loss upon SETDB1 loss raises the possibility that SETDB1 could constitute an exciting target for therapy in ALT, perhaps through the use of a small molecule inhibitor.

## Altered rDNA Heterochromatin in ATRX-Deficient Cancers

Along with telomere heterochromatin changes in ALT, recent evidence has suggested a role for the binding of ATRX to another of its highly repetitive binding sites—ribosomal DNA (rDNA). The work, in mouse ES cells, demonstrates the importance of ATRX and DAXX in the deposition of histone H3.3 into ribosomal repeat sequences and, in the absence of effective deposition, cells progressively encounter rDNA copy number alterations and rDNA repeat instability. Following ATRX loss, the reduction in rDNA copy leads to proportionally reduced ribosomal RNA (rRNA) transcription, which ultimately results in increased sensitivity to inhibitors of the RNA Polymerase PolI (the principle polymerase for rRNA). The authors go on to demonstrate that canonical ALT cells, likely due to their lack of functional ATRX, are equally sensitive to PolI inhibitors, suggesting that PolI inhibitors could be effective on a wide range of ALT cancers (52).

## APBs and Their Formation

APBs are, as previously mentioned, a marker of ALT and are considered to be a major site of telomere recombination in ALT. Following the generation of a DSB at telomeres, evidence suggests that telomeres migrate rapidly and cluster within APBs in a RAD51-dependent manner, awaiting downstream processing (53).

Work has previously shown that APBs are required for ALT and that overexpression of the nuclear autoantigen Sp-100, a constituent component of PML bodies, inhibited APB formation through the sequestration of the MRN complex component NBS1, another constituent component of APBs. This sequestration ultimately led to progressive telomere length shortening and a reduction in telomere length fluctuations (54). Along with Sp-100, a number of other proteins are required for effective APB formation including PML, TRF1 and TRF2, and the SUMO E3 ligase MMS21. The latter, MMS21 is part of the SMC5/6 complex and is considered to be an essential SUMO E3 ligase in the generation of APBs, with its primary role in SUMOylating TRF1 and TRF2 as part of APB formation. In studies where the SUMO target sites in TRF1/2 are disrupted, or the SUMO-ligase dead mutant of MMS21 was introduced into an MMS21 null cell line, APB formation was severely impaired (55).

In part owing to the complexity of APB formation, and their intrinsic requirement for the ALT process, APBs and their formation are a strong candidate for the development of therapies. One such therapy could be a SUMO E3 ligase inhibitor, or inhibitors of the SENP family of proteins, proteins that cleave the inactive precursor form of SUMO and catalyze the de-conjugation of SUMO to its target protein (56).

## Break Induced Replication Drives ALT Telomere Synthesis

Telomere maintenance in ALT is thought to be mediated by a pathway referred to as break induced replication (BIR), with a preference for lagging strand synthesis (57). Seminal work in the field from Dilley et al. implicated a primary three protein axis of POLD3, PCNA, and RAD52 in the mechanism of ALT telomere extension. In this work the authors demonstrate that ALT telomere maintenance is independent of RAD51, a protein responsible for homology search in homologous recombination, and is instead dependent on the less well-defined paralog RAD52. The reliance on RAD52 over RAD51 is an interesting observation, due to the involvement of RAD51, together with the Hop2-Mnd1 heterodimer in the facilitation of long-range telomere migration as well as the colocalization of these proteins to APBs in ALT cells (58). However, yeast studies have shown both RAD51-dependent and -independent mechanisms of BIR in telomere maintenance exist, highlighting the possibility that BIR in humans may occur in a similar fashion (59).

Additional ancillary factors are recruited to telomeres in ALT and have been hypothesized to play a number of roles. One such factor, FANCD2, a component of the Fanconi anemia complex, is both recruited to CFS with its partner protein FANCI and also to telomeres in ALT positive cells (60, 61). FANCD2, in ALT cells, appears to act in opposition to the Bloom Syndrome Helicase (BLM) to restrain telomere replication and recombination, and its depletion leads to a hyper-ALT phenotype. Depletion of FANCD2 alone, however, does not trigger the ALT phenotype, indicating the involvement of additional factors. Increased telomeric DNA content as a consequence of telomere elongation and tSCEs in FANCD2-depleted cells occur through a RAD51-independent mechanism, which one could hypothesize is in agreement with the notion of a RAD51-independent, RAD52-mediated ALT process (62). Additionally, the recent observation of MiDAS at telomeres, has also been shown to be RAD52-dependent and SLX4-dependent but RAD51-independent (22, 63). Although, work by Sobinoff et al. shows an opposing role for SLX4 in ALT telomere elongation and as such, further work will need to be done to determine and fully characterize its role in controlling BIR (64).

## ALT is a Bifurcated Pathway

Recent work has built upon the previous observation of RAD52-dependent BIR-mediated telomere extension in ALT to further distinguish the mechanism of ALT. Alongside RAD52-dependent BIR, compelling evidence exists for a second RAD52-independent mechanism of ALT. In this case, the commonly held phenotypical markers of ALT, APBs, and C-circle generation, can be considered to be a consequence of a bifurcated ALT pathway. Indeed, when Zhang et al. investigated RAD52 knockout (RAD52Δ) ALT cells it was noted that while in the initial period following RAD52Δ telomeres rapidly shortened, presumably as a consequence of a loss of telomeric BIR, telomere length subsequently stabilized. The authors were able to show that telomere synthesis in APBs was acutely dependent on RAD52. In contrast, RAD52 was dispensable for C-circle generation, yielding no immediate reduction in C-circles in RAD52Δ cells (65).

After telomeres reached considerably short lengths, however, an alternative, RAD52-independent, ALT pathway emerged, showing increased C-circle levels and telomere synthesis at APBs. This RAD52-independent pathway was indeed still dependent on POLD3 and its partner POLD4, as well as BLM, as evidenced by a loss of DNA synthesis in APBs and C-circles. Additionally, despite recapitulating ALT markers such as C-circles and APBs, telomere length was not maintained to the same degree as when the cells utilized a RAD52-dependent mechanism. This suggests a fundamental difference in the two mechanisms, a difference which could be attributed to the observation by the authors of the ability of RAD52 to initiate telomeric D-loop formation, a critical step in BIR, even in the presence of Replication Protein A (RPA) coated ssDNA (65). Additionally, loss of the structure specific endonuclease scaffold SLX4 reduces proliferation in ALT cells lacking RAD52Δ, with some evidence suggesting the existence of an SLX4-dependent, but RAD52-independent mechanism of telomere stability (66).

These data suggest that canonical “ALT” may indeed be an amalgamation of multiple similar but distinct pathways, the first being RAD52-dependent, and the second being RAD52-independent, with the RAD52-dependent pathway contributing to the majority of the telomere extension, and the RAD52-independent pathway acting as an alternative pathway that recapitulates some ALT markers, but with reduced efficiency. Nevertheless, more work is needed to fully decipher these intertwined pathways.

## The Role of ATR and ATM in ALT

Recent work has demonstrated that ALT-associated BIR is independent of the ataxia telangiectasia-mutated (ATM) protein kinase and ataxia telangiectasia and Rad3-related protein (ATR), both of which are key regulators of DNA damage repair, and the initial damage is instead sensed by an replication factor C (RFC) and Proliferating cell nuclear antigen (PCNA) (14). Previous work, however, suggests that ALT cells display sensitivity to ATR inhibitors (67). In this work, the authors demonstrate a reduction in ALT markers including APBs, tSCEs, and C-circles in the presence of the ATR inhibitor VE-821 and a reduction in APBs upon knockdown of ATR with siRNA. The authors propose this is a consequence of an accumulation of DNA damage, increased micronuclei formation and aberrant anaphase chromosome segregation. ALT cells, when exposed to the ATR inhibitor VE-821, show significantly reduced survival and a considerably lower IC50. In corroboration with Dilley et al. and Flynn et al. demonstrated that ALT cells lacked any sensitivity to ATM inhibitors (67). Subsequent studies, however, have failed to identify a sensitivity, calling into question the initial finding of using ATR inhibitors in treating ALT cancer (68).

Together, these data suggest that it is unlikely that ALT relies on an ATM or ATR-modulated pathway for telomere extension, and instead likely relies on RFC and PCNA. This distinction is important to note in regard to therapeutic treatment of cancers with ATR inhibitors, which in this case may not improve outcomes. One may hypothesize that, in fact, ATR inhibition may lead to defects in ssDNA break repair or fork stalling, which may in turn exacerbate the ALT phenotype.

## NuRD-ZNF827—A Driver of ALT Recombination

Another characteristic marker of ALT is the presence of telomere variant repeats interspersed throughout the telomere (69). These repeats are thought to occur as a consequence of subtelomeric recombination, or rare nucleotide misincorporation by telomerase, and levels of variant repeats vary between different ALT cancers (70, 71). Shelterin components, evolutionarily adapted to binding to canonical repeats, display lower affinity to these variant repeats, allowing for the binding of the NR2C/F class orphan nuclear receptors (72). Orphan receptors in this class, including TR2, TR4, and COUP-TF2 have been shown to recruit the zinc finger protein ZNF827 to ALT telomeres and provide a platform for the recruitment of the nucleosome remodeling and histone deacetylation (NuRD) complex. The NuRD complex, in turn, has been shown to promote telomere-telomere recombination through binding together telomeres from different chromatids or chromosomes, recruitment of HR factors, as well as displacement of Shelterin components and induce further replicative stress. Together, NuRD and ZNF827 can be considered as potent drivers of the ALT process, however, the HDAC properties of NuRD also has protective properties, including counteracting excessive de-heterochromatinisation and a buffering effect on telomere bridge formation (73). Recent progress has been made in the development of histone deacetylase (HDAC) inhibitors which could be used to selectively inhibit ALT through NuRD suppression. Unfortunately, it is still unclear as to whether HDAC inhibitors could be selective enough, with potential effects, in non-cancerous tissues (74).

## RPA and Telomere Transcription in ALT

Many of the aforementioned ALT processes, in part, lead to the formation of either ssDNA overhangs or regions of ssDNA. *In vivo*, ssDNA is rapidly coated by RPA, which serves to both protect ssDNA from the formation of secondary structures, as well-facilitate many aspects of DNA repair including activation of ATR signaling pathways (75). It has been proposed that release of RPA from telomeres may be an important mechanism to suppress HR, and thus BIR, at telomeres (67).

Regulation of RPA at telomeres would thus be essential to ensure appropriate repair of telomeric damage. Indeed, work by Flynn et al. has proposed one such mechanism of RPA regulation at telomeres. The authors propose the involvement of the telomeric repeat containing RNA (TERRA), a long non-coding RNA (lncRNA) that ranges from 100 to 9 kb and is thought to be involved in many telomeric processes, in the sequestration of RPA from telomere ends in a cell cycle dependent manner (67, 76).

In order to prevent ATR signaling at telomeric overhangs, such as those found at T-loops, the Shelterin components POT1 and TPP1 must prevent RPA binding. POT1-TPP1, however, is only able to displace a minor fraction of telomere-bound RPA in *in vitro* assays, and instead cells rely on displacement of RPA by hnRNPA1, which is unable to displace POT1. It is then proposed that hnRNPA1 activity is itself mediated by TERRA transcript levels, which vary throughout the cell cycle (77).

ATRX loss is proposed to lead to an accumulation of TERRA in late-S/G2, whereas in ATRX wild type cells TERRA levels are high in S phase only, with multiple studies indicating higher overall TERRA levels in ALT and ATRX-null cells (67, 78). This accumulation of TERRA then prevents the release of RPA from telomeric ssDNA by hnRNPA1, which in turn leads to ATR signaling and a DNA damage response. This phenotype is marked by the appearance of large damage related RPA foci in G2/M specifically in ALT positive cells and non-ALT cells lacking ATRX (67).

Along with a proposed role in RPA sequestration from telomeres, TERRA has been implicated in the promotion of telomeric heterochromatin expansion, through its binding to both HP1 and the H3K9me3 (79). Additionally, TERRA is thought to be crucial for the short-term protection of T-loops from DNA helicases through the formation of DNA:RNA hybrid structures at telomere ends, termed R-loops (78, 80). These telomeric R-loops could hypothetically pose significant threat to efficient telomere replication.

## DNA Secondary Structures and R-loops in ALT

An inherent feature of telomeres is their ability to adopt non-canonical secondary structures including G-tetrad structures called G-quadruplexes (G4). Previous work has suggested that ATRX binds to these G4 structures (81), and has a potential role in their resolution, with ATRX-null cells displaying higher numbers of G4 structures (81, 82). Consistent with this notion, recent work has shown that in an ATRX-null background cells are unable to effectively tolerate the induction of these structures through chemical stabilization, raising the possibility for their use in either selectively killing ATRX-null ALT cells, or in pre-sensitizing these cells to other therapeutic agents such as ionizing radiation (82, 83). A number of G4 stabilizers are currently in clinical trials, with one example being CX-3543 (Quarfloxin) (84). Additionally, it has been reported that introduction of the G4 stabilizing ligand PDS into cells induces MiDAS, with a significantly stronger effect in ALT positive cells. Taken together this infers that the presence of G4 structures may potentiate ALT but also may offer a therapeutic target (22).

R-loops are composed of a three-stranded nucleic acid structure, where the nascent RNA forms a hybrid with the DNA template strand and displaces the non-template DNA strand. R-loops are also thought to occur in regions within the genome that are enriched in guanine nucleotides and are therefore highly coincident with G4 forming regions (85). R-loops also have the potential to be major contributors to genome instability and are thought to cause replication fork stalling, collapse and generation of DSBs if they remain unresolved prior to replication (86). Strikingly, ALT cancer cells have been reported to have higher levels of R-loops and binding of RNaseH1 (an enzyme that degrades RNA:DNA hybrids) at telomeres. Significantly, overexpression of RNaseH1 attenuates ALT markers, suggestive of a role of R-loops in potentiating ALT (78).

In addition to RNaseH1, FANCM, and ATRX have been shown to have roles in regulating R-loops in the context of ALT (78, 87–89). Recent published literature suggests FANCM is important for replication fork remodeling and DNA damage repair and resolves R-loops at the telomeres. The authors show that FANCM is recruited to telomeres and in its absence, there is an accumulation of telomeric RNA:DNA hybrids. Moreover, overexpression of RNaseH1 suppressed the enhanced ALT markers present after FANCM depletion. This therefore suggests that R-loop formation has a potential role in generating the replication stress needed for ALT initiation/exacerbation. In addition, the authors demonstrated that the ATPase/translocase domain of FANCM was responsible for R-loop resolution, as mutations in this domain generated increased R-loops (88).

ALT cancer cells have been suggested to have increased TERRA that is thought to act in cis or trans to form R-loop structures at the telomeres (78). Silva et al. showed that with FANCM depletion, TERRA transcript levels increased significantly, implicating FANCM in the modulation of R-loop formation by also controlling TERRA. FANCM mediated suppression of ALT has additionally been shown to be dependent on its interaction with the BLM-TOP3a-RMI (BTR) complex and disruption of this interaction using the PIP-199 small molecule inhibitor has been shown to be selectively toxic to ALT cancer cells (88).

## Translesion Synthesis in ALT

Replication stress, whether it be caused by exogenous agents or structures such as R-loops and G-quadruplexes, must be dealt with to avoid replication fork collapse. We have previously discussed the role of ATRX in replication fork protection, as well as the consequences of failure to protect these forks. Recent work has implicated another DNA repair network in the protection of ALT telomeres.

Using BioID, a technique that utilizes proximity-dependent biotinylation, Garcio-Exposito et al. revealed proteins associated with telomeres in both an ALT and non-ALT context (90). In both ALT and non-ALT samples this method detected common telomere-associated proteins, such as the Shelterin complex as well as several Shelterin accessory proteins including MRE11, BLM, PARP1, and Tankyrase 1. In ALT positive samples, the method pulled out many ALT associated proteins including PML, ERCC1 and SLX4, providing validation for the technique.

In addition to these ALT-specific factors, however, the authors also found enrichment of factors that functionally converge to regulate RAD18-mediated mono-ubiquitination of PCNA during translesion DNA synthesis (TLS), namely FANCJ, RAD18, and the specialized Y-family polymerase DNA Polη. TLS is a mechanism of DNA repair that allows replication machinery to bypass lesions by replicating directly over them with the aid of specialized polymerases that are amenable to distorted templates (91). Following depletion of one such polymerase, Polη, APBs and C-circles increased in ALT cells, indicating a link between Polη and the ALT process. After induction of specific breaks in telomeres, Polη knockdown did not lead to differential ALT marker output, indicating that Polη does not directly operate within the ALT pathway itself.

These data could indicate a role for Polη, and the TLS pathway, in the protection of stalled telomeric replication forks in ALT, attempting to prevent collapse of forks that may lead to BIR. The observation of SLX4 at ALT telomeres lends credence to this hypothesis, due to the described role of SLX4 in promoting HDR through fork collapse. In this regard, the authors demonstrate that HDR at ALT telomeres increases in the absence of Polη (90).

An open question remains as to what is the cause and origin of potential telomeric lesions in ALT cancer cells that require the TLS machinery for replication and repair. Previous studies have shown that, in response to loss of telomerase, ALT tumors showed increased levels of mitochondrial reactive oxygen species (ROS), and upregulation of core mitochondrial oxidative defense genes including PGC-1β, and its targets such as NRF2, SOD2, and Catalase (92). Such an increase in ROS could induce oxidative damage at telomeres, and lead to the generation of 8-oxodG within the GGG triplet of telomeres, as previously described, which would thus be targets of a TLS repair pathway (93). Along with providing insights into one potential mechanism of ALT generation, Hu et al., demonstrated the susceptibility of ALT positive cells to PGC-1β or SOD2 knockdown, suggesting that the development of small molecule inhibitors of this pathway could provide an exciting future therapy for ALT (92).

## Alternative Mechanisms of Dealing with Replication Stress in ALT

Along with TLS as a mechanism of reducing the effects of replicative stress, literature evidence exists for the involvement of the replication stress response protein SMARCAL1 in the regulation of ALT activity (94). SMARCAL1 is a 954-amino acid protein containing an RPA binding domain at the N-terminus (95). Alongside its role as a DNA strand annealing helicase, SMARCAL1 catalyzes fork reversal in response to replication stress that causes replication fork stalling (96–98). This fork reversal stabilizes the replication fork, allowing for processing of the fork and attempted resolution of the cause of the replication stress.

A study in ALT positive glioblastoma found that SMARCAL1 mutations, like those of ATRX and DAXX, correlate well with ALT status, with over half of ALT positive glioblastoma samples harboring SMARCAL1 mutations (99). Interestingly, the authors also note that these mutations are largely mutually exclusive with ATRX mutations, implying that both ATRX mutations and SMARCAL1 mutations may ultimately lead to the same outcome. Consistent with a role in protecting against ALT, SMARCAL1 depletion augments C-circles as well as markers of telomeric DNA damage in ALT cells (94, 100).

Additionally, loss of the Anti-Silencing Factor 1 paralogs ASF1a and ASF1b, histone chaperones that assist in the transfer of H1.3-H4 or H3.3-H4 histone dimers to the CAF-1 and HIRA proteins respectively, have been described to trigger an ALT-like phenotype. Depletion of ASF1a in a long telomere HeLa background, without any concomitant mutations in factors such as ATRX, was sufficient to trigger both APBs and C-circles, likely also through the generation of replication stress (101).

In addition to chromatin remodeling, cells have a number of other ways to protect replication fork progression. One such complex of proteins, the fork protection complex (FPC), is composed of the TIMELESS and TIPIN proteins (102). Recent studies have indicated that the TIMELESS/TIPIN, protect telomeres from replication stress by suppressing break-induced replication processes (103). Analogous to SMARCAL1 depletion of TIMELESS or TIPIN in an ALT cell leads to an increase in ALT markers, including telomere clustering, APB formation and telomeric MiDAS (22). Taken together these studies once more reinforce the need for cells to protect against telomeric replication stress in order to prevent induction of ALT.

## Other Treatment Possibilities for ALT

Along with the previously mentioned treatment opportunities in ALT, the loss of ATRX/DAXX in a vast majority of ALT cancers presents a unique opportunity for therapy. It has long been established that, outside of its role as a chromatin remodeller, ATRX has a role in the innate viral immune response. Previous work has shown that many viruses contain protective mechanisms to repress ATRX mediated viral responses. In the case of herpes simplex virus (HSV), during early viral infection viral particles are localized adjacent to discrete nuclear structures known as nuclear domain 10 (ND10), of which ATRX and DAXX are constituent components (104–106). In response to this, HSVs enlist the activity of the immediate early (IE) protein ICP0, which disperses the ND10 compartment and degrades PML (107–109). ICP0-null viruses, on the other hand, are largely unable to replicate within ATRX positive cells (110). Thus, an exciting avenue of potential therapy would be to use ICP0-null viruses to deliver a fatal payload to ATRX-null cells, a so-called oncolytic virus approach (111).

## Conclusion

**Figure 1:**
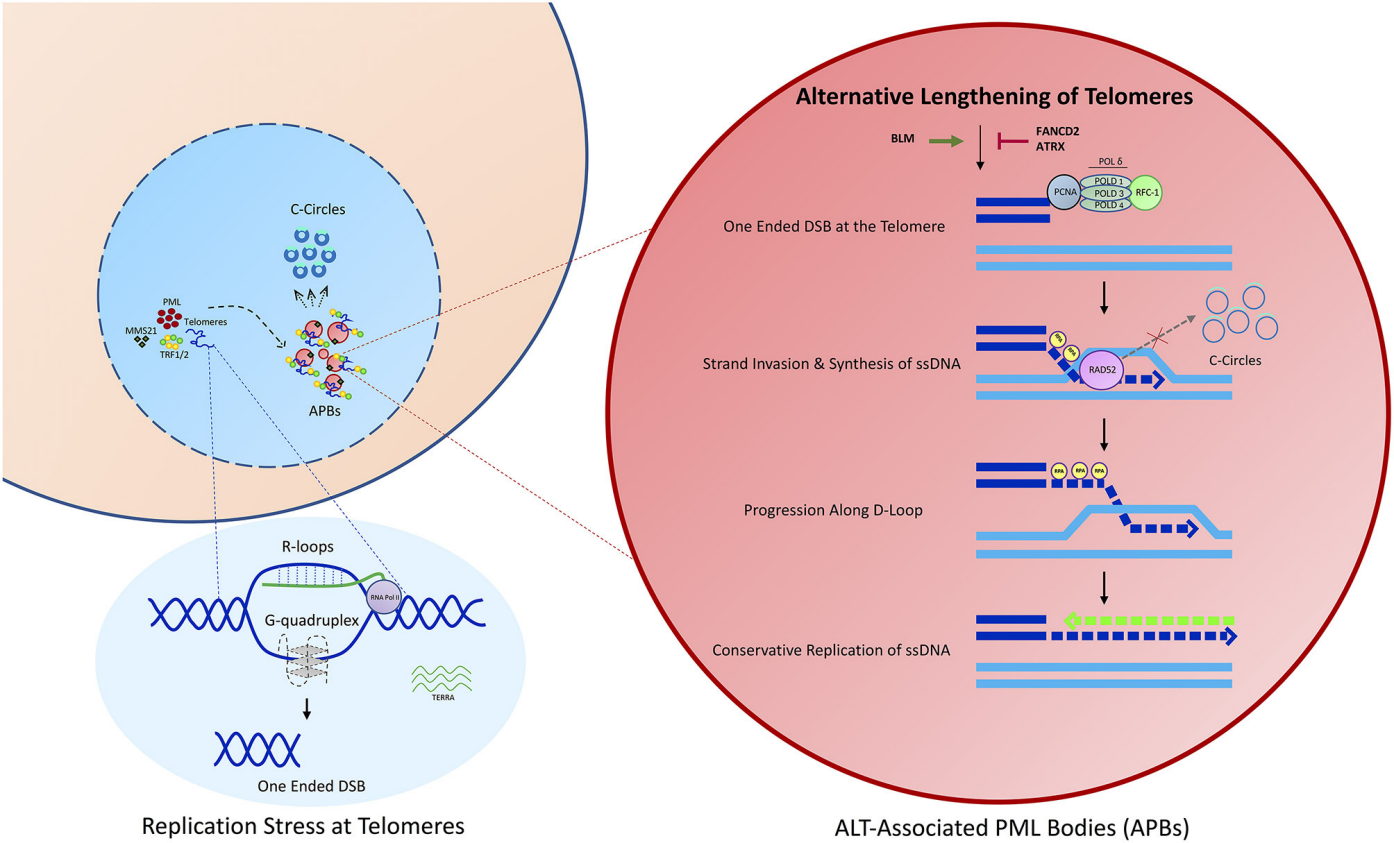
An overview of the ALT process. Telomeres in ALT cancer cells undergo replicative stress potentially as a result of DNA secondary structure formation, including R-loops and G-quadruplexes. This results in the formation of a one ended double strand break. Damaged telomeres are clustered into ALT associated PML nuclear bodies, potentially mediated through the SUMOylation of Shelterin components, including TRF1 or TRF2. APBs constitute the site of recombination where telomeres are extended predominantly via a process of Rad52 dependent Break Induced Replication (BIR).

Over the last few years much progress has been made in elucidating the underlying mechanism behind ALT, however much work remains to be done (Figure 1). Questions still linger over the chronology in which different components of the ALT pathway are activated, and what was once a single mechanism of ALT telomere extension is now understood to be multiple related pathways. As a result, additional research is needed to fully elucidate the mechanism of ALT, and with each characterized pathway, additional therapeutic targets become clear. To summarize a list of potential targets and therapies for the treatment of ALT cancers discussed in this review is shown in Table 1.

**Table 1:** Potential targets and therapies for the ALT pathway.

**Target**	**Rationale**
ATR inhibitors	Conflicting evidence of susceptibility of ALT cells to ATR inhibitors, evidence in the literature of susceptibility to VE-821 (67)
Rad52 inhibitors	RAD52-mediated BIR facilitates ALT but has few other functions within most cells making it a unique target in ALT (14)
PolI Inhibitors	rDNA copy number loss upon loss of ATRX sensitizes ATRX-null ALT cells to PolI inhibitors such as CX5461 (52)
Oncolytic Viruses	ATRX and DAXX work in concert to protect cells from viral invasion, leaving ALT cells susceptible to ICP0-null viruses (110)
HDAC inhibitors	Evidence of the involvement of the NuRD complex may indicate efficacy of HDAC inhibitors in ALT (73)
G4 Stabilizer	Work has shown ALT cells are susceptible to excessive G4 stabilization using ligands. Examples include: Pyridostatin, Phen-DC3, CX-3543 (22)
SETDB1 inhibitors	SETDB1 loss leads to a reduction in ALT markers (51)
PGC-1β/SOD2 inhibitors	Members of the core mitochondrial oxidative response lead to increase mitochondrial ROS which may be a trigger of ALT (92)
SUMO E3 Ligase/SENP inhibitors	SUMOylation is essential for the generation of APBs, which are in turn essential for ALT telomere lengthening (55)
DNA damaging agents	ATRX-deficient cells display reduced ability to repair DNA DSBs generated with compounds such as MMS and MMC (39)

## Author Contributions

TK and DC: planning, writing, and critical reading of manuscript. DG: writing of manuscript. SS: contributed Table 1 and critical reading of manuscript.

### Conflict of Interest

The authors declare that the research was conducted in the absence of any commercial or financial relationships that could be construed as a potential conflict of interest.
